# Impact of Prevalence Ratios of Chondroitin Sulfate (CS)- 4 and -6 Isomers Derived from Marine Sources in Cell Proliferation and Chondrogenic Differentiation Processes

**DOI:** 10.3390/md18020094

**Published:** 2020-01-31

**Authors:** Estefanía López-Senra, Paula Casal-Beiroa, Miriam López-Álvarez, Julia Serra, Pío González, Jesus Valcarcel, José Antonio Vázquez, Elena F. Burguera, Francisco J. Blanco, Joana Magalhães

**Affiliations:** 1 New Materials Group, Department of Applied Physics, University of Vigo, IISGS, MTI, Campus Lagoas-Marcosende, 36310 Vigo, Spain; eslopez@uvigo.es (E.L.-S.); miriammsd@uvigo.es (M.L.-Á.); jserra@uvigo.es (J.S.); pglez@uvigo.es (P.G.); 2 Unidad de Medicina Regenerativa, Grupo de Investigación en Reumatología, Instituto de Investigación Biomédica de A Coruña (INIBIC), CHUAC. SERGAS. C/ As Xubias de Arriba 84, 15006 A Coruña, Spain; paula.casal.beiroa@sergas.es (P.C.-B.); elena.fernandez.burguera@sergas.es (E.F.B.); fblagar@sergas.es (F.J.B.); 3 Centro de Investigaciones Científicas Avanzadas (CICA), Universidade da Coruña (UDC), As Carballeiras S/N, Campus de Elviña, 15071 A Coruña, Spain; 4 Grupo de Reciclado y Valorización de Materiales Residuales (REVAL), Instituto de Investigacións Mariñas (IIM-CSIC), Eduardo Cabello 6, 36208 Vigo, Spain; jvalcarcel@iim.csic.es (J.V.); jvazquez@iim.csic.es (J.A.V.); 5 Centro de Investigación Biomédica en Red (CIBER), Av. Monforte de Lemos, 3-5. Pabellón 11, 28029 Madrid, Spain; 6 Departamento de Medicina, Facultad Ciencias de la Salud, Campus de Oza, Universidade da Coruña (UDC), Campus de Oza, 15006 A Coruña, Spain

**Keywords:** chondroitin sulfate, osteoarthritis, chondrogenesis, fishery by-products, marine compounds, circular economy

## Abstract

Osteoarthritis is the most prevalent rheumatic disease. During disease progression, differences have been described in the prevalence of chondroitin sulfate (CS) isomers. Marine derived-CS present a higher proportion of the 6S isomer, offering therapeutic potential. Accordingly, we evaluated the effect of exogenous supplementation of CS, derived from the small spotted catshark (*Scyliorhinus canicula*), blue shark (*Prionace glauca*), thornback skate (*Raja clavata*) and bovine CS (reference), on the proliferation of osteochondral cell lines (MG-63 and T/C-28a2) and the chondrogenic differentiation of mesenchymal stromal cells (MSCs). MG-G3 proliferation was comparable between *R. clavata* (CS-6 intermediate ratio) and bovine CS (CS-4 enrichment), for concentrations below 0.5 mg/mL, defined as a toxicity threshold. T/C-28a2 proliferation was significantly improved by intermediate ratios of CS-6 and -4 isomers (*S. canicula* and *R. clavata*). A dose-dependent response was observed for *S. canicula* (200 µg/mL vs 50 and 10 µg/mL) and bovine CS (200 and 100 µg/mL vs 10 µg/mL). CS sulfation patterns discretely affected MSCs chondrogenesis; even though *S. canicula* and *R. clavata* CS up-regulated chondrogenic markers expression (aggrecan and collagen type II) these were not statistically significant. We demonstrate that intermediate values of CS-4 and -6 isomers improve cell proliferation and offer potential for chondrogenic promotion, although more studies are needed to elucidate its mechanism of action.

## 1. Introduction

Osteoarthritis (OA) is the most prevalent rheumatic disease and a leading cause of chronic pain, associated with increased rates of comorbidity and one of the most burdensome disabilities worldwide [1]. This multifactorial pathology affects all joints and is characterized by cellular stress and degradation of the hyaline articular cartilage [2]. No cure for OA has been found to date and most treatments available are directed to reduce pain and/or inflammation [3]. Indeed, the main goal of OA therapy should be oriented to delay cartilage degeneration and promote regeneration.

Due to its unique properties, sulfated glycosaminoglycans (GAGs), found in high abundance in mammals and invertebrates have received special focus for OA applications [4]. The treatment with chondroprotectives, such as glucosamine, hyaluronic acid and chondroitin sulfate (CS) has been extensively reported and these have shown, in several *in vitro* and *in vivo* studies, their effect in modifying, stabilizing, retarding or even reversing the pathology of OA [5,6,7,8]. Even though in the early 2000s CS was given the highest evidence grade and the highest recommendation grade concerning knee OA [9], its mechanism of action is still to be elucidated and its efficacy remains under debate.

Chondroitin sulfate (CS), as a natural component of the extra-cellular matrix (ECM), is a sulfated GAG that consists of repeating disaccharide units of N-acetylgalactosamine (GalNAc) and glucuronic acid (GlcA), joined by β1,4 and β1,3 linkages, respectively. Most CS contain sulfate groups in either the 4- (CS-A) or 6-position (CS-C) of the GalNAc unit, but may also be sulfated at both (CS-E), making CS a strongly charged polyanion [10]. Several sulfotransferases (STs) involved in 4-O- and 6-O-sulfation of GalNAc units are responsible for obtaining CS with different degrees of sulfation (0.1–1.3 per disaccharide unit) and patterns [4].

Moreover, several authors have suggested different biological roles of CS within cartilage biology, such as signaling functions of various growth factors that are closely associated with the sulfation patterns of CS. In addition, mutations in STs genes cause cartilage and bone abnormalities, which highlight the importance of CS sulfation in chondrogenic differentiation [11]. CS sulfation patterns have also been shown to be implicated in the proliferation of chondrocytes, modulation of chondrogenesis in primary mesenchymal stromal cells (MSCs) or the ATDC5 prechondrogenic cell line, either as an exogenous factor supplemented in the medium or embedded in drug-delivery systems, as well as being a component of scaffolds [12,13,14,15].

Differences have also been found in the prevalence of CS isomers in the onset of osteoarthritis. A decrease in the ratio of CS-6 to CS-4 has been described in advanced stages of OA, both in full-thickness human articular cartilage and synovial fluid samples [5,16,17]. Altogether, these changes suggest that regulation of GAGs synthesis may be useful in the treatment of cartilage disorders.

External sources for supplementation with higher proportion of CS-6 are rare, but the discovery of sustainable sources derived from fishery by-products, due to a boost in circular economy research and innovation policies, offers great potential for OA applications [18]. Recently, CS from cartilaginous fish by-products and discards have been characterized as valuable sources of predominantly CS-6 isomer, available in high yields and large volumes from the fish-processing industry [19,20,21].

These comprehensive works were further complemented with a quantitative evaluation by Raman spectroscopy, elucidating differences amongst the different CS sources, using mammalian—bovine source—commercially available CS as reference [22]. According to previous results found in the literature, the differences described could be attributed to either a greater prevalence of the isomer GlcA-GalNAc 6S in blue shark (*Prionace glauca*) and thornback skate (*Raja clavata*) CS (% GlcA-GalNAc 4S / % GlcA-GalNAc 6S, also designated as C4S/6S of 0.16 and 0.29, respectively), or the isomer GlcA-GalNAc 4S in the case of commercial bovine CS (C4S/6S of 1.47). On the other hand, small spotted catshark (*Scyliorhinus canicula*) CS presented comparable contribution of both isomers (C4S/6S of 0.97), according to data obtained by strong anion exchange-high performance liquid chromatography (SAX-HPLC) and other techniques [19,23,24].

Finally, validated indices of major 4S or 6S isomeric contributions, based on calculated ratios from Raman molecular vibrations of the main skeletal modes (Table 1), were proposed for the rapid discrimination of the major contribution of C6S or C4S in a given sample [22]. In the present work, we aimed at studying biological responses to the exogenous supplementation of CS derived from *S. canicula, P. glauca* and *R. clavata*, enriched in either 4S or 6S isomers by (1) evaluating cell viability and proliferation using osteoblastic and chondrocytic cell lines, and (2) evaluating their potential as chondrogenic promotors using mesenchymal stromal cells derived from the bone marrow of OA donors.

## 2. Results and Discussion

CS derived from cartilaginous fish has been described as a potential source of 6S-enriched CS. In previous studies, we thoroughly described the molecular composition of *Scyliorhinus canicula*, *Prionace glauca* and *Raja clavata* through Raman spectroscopy, and other techniques, supporting similarities in their structures but also main alterations in the characteristic bands according to the species [19,22]. In this work we tested the effect of the different sources of CS and consequent CS-4 to -6 ratios contribution in the proliferation of an osteoblastic (MG-63) and a chondrocytic (T/C-28a2) cell lines and evaluate possible dose-dependent effects. Cell lines hereby reported are both representative of osteoblasts and chondrocytes present at the osteochondral interface, and its relevance could be considered when envisioning future applications of CS in regenerative medicine that include full thickness cartilage defects.

### 2.1. Viability in MG-63 Cell Line

**Figure 1:**
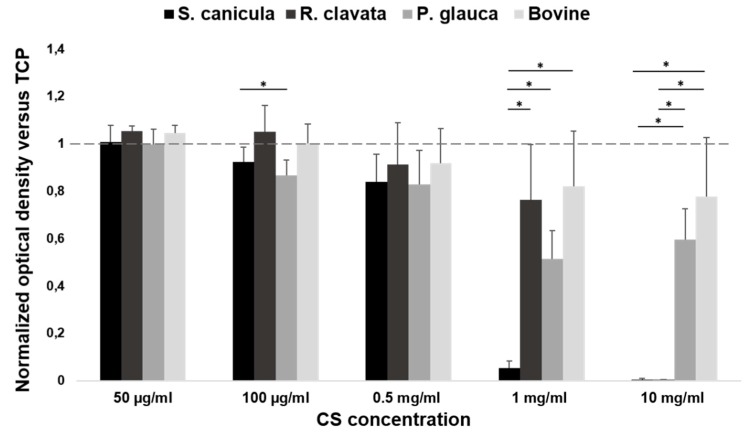
MG-63 osteoblast cell line proliferation (MTT assay), after 72 h incubation in minimum essential medium-Eagle with Earle’s balanced salt solution (EMEM) supplemented with different concentrations of chondroitin sulfate (CS) (50 µg/mL, 100 µg/mL, 0.5 mg/mL, 1 mg/mL and 10 mg/mL), from fish (*Prionace glauca*, *Raja clavata* and *Scyliorhinus canicula*) and bovine sources, normalized against CS-free condition which was assigned the value of 1. Significant statistical differences for *p* < 0.05 (*) are shown.

The viability of the osteoblastic-like cell line MG-63 exposed to different concentrations of CS (50 µg/mL to 10 mg/mL) supplemented to the cellular growth medium is depicted in Figure 1. Absorbance results were normalized against cells cultured on CS-free (growth medium) condition. The dotted line represents a ratio of 1, which indicates the same cellular viability as in CS-free. After 72 h incubation, results revealed an absence of cytotoxicity for CS concentrations below 0.5 mg/mL. Given that, 0.5 mg/mL could be considered as a threshold concentration of CS for MG-63 cell line, as cellular viability is guaranteed for equal and lower concentrations. Higher doses revealed values close to zero for *S. canicula* and *R. clavata* CS, with normalized values significantly lower regarding CS-free viability, which presented mean values below the dotted line and high variability between replicates for the same CS. At 0.5 mg/mL CS concentrations, cell viability presented similar values for all CS sources and the mean values were below the dotted line. It is at the concentration of 100 µg/mL, where optical density of CS obtained from *R. clavata* overcomes the dotted line and, therefore, favors MG-63 cell viability in relation to CS-free conditions. This effect was significantly higher (p < 0.05) than the viability obtained for *P. glauca* CS, and superior to *S. canicula* and bovine CS. Finally, the lowest concentration (50 µg/mL) revealed benefit in mean value of CS from *R. clavata* and bovine CS, in comparison to CS-free, and the same viability observed for *P. glauca* and *S. canicula* CS, but no statistical significant improvement was found, for any of the tested sources at this concentration. 

Considering the physicochemical characterization by Raman spectroscopy [22], it was possible to establish ratios that allow the classification of the different types of chondroitin sulfate of piscine and bovine origin as major sulfation 6S, 4S or equal contribution. Although none of the CS sources tested were cytotoxic for MG-63 cells, when concentrations below the threshold of 0.5 mg/ml were tested, we could not find a specific trend between cells’ proliferation and the potential benefit of 4S or 6S enrichment. Nonetheless, it has been shown that chondroitin sulfate (specifically commercial CS, 4S) influences *in vitro* proliferation of osteosarcoma cells and this could be associated to the binding of CS to growth factors present in the environment or produced by the cells, increasing their bioavailability [25]. In our case, the proliferation of osteoblasts for concentrations below 0.5 mg/mL is comparable to that of commercial bovine CS (4S) as well as in CS obtained from *R. clavata* (6S) or even slightly lower in mean value, which again confirms the absence of influence of a majority isomer (CS-4 or CS-6) on the proliferation of the MG-63 line.

### 2.2. Viability and Proliferation in T/C-28a2 Cell Line

**Figure 2:**
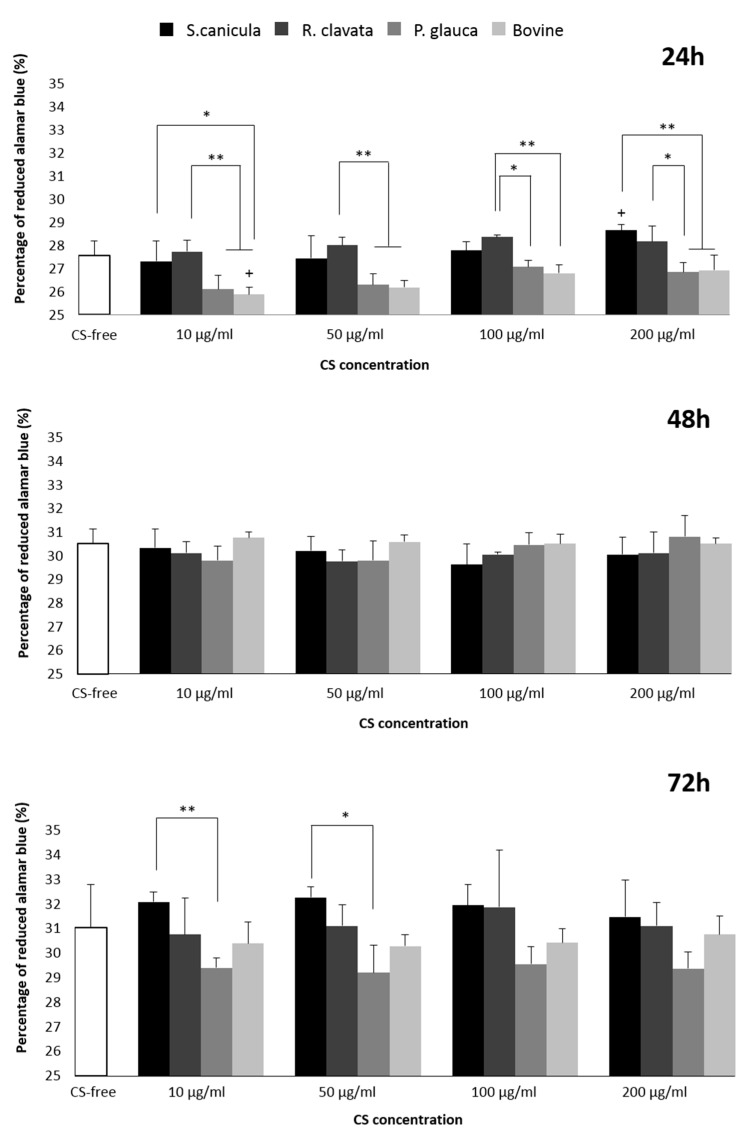
T/C-28a2 chondrocytic cell line viability and proliferation (directly proportional to alamar blue dye reduction) after 24, 48 and 72 h incubation in basal medium (Dulbecco’s modified Eagle’s medium (DMEM) 10%) supplemented with different concentrations of CS (10 µg/mL, 50 µg/mL, 100 µg/mL and 200 µg/mL) or CS-free, derived from fish (*Prionace glauca*, *Raja clavata* and *Scyliorhinus canicula*) and bovine sources. Significant statistical differences for *p* < 0.05 (*) and *p* < 0.01 (**) are shown. No significant differences were found amongst the different CS sources and CS-free condition.

We further studied the effect of different sources of CS in the viability and proliferation of T/C-28a2, a chondrocytic cell line, for 24, 48 and 72 h, as represented in Figure 2. As the 100 µg/mL concentration was described as being below the threshold for MG-63, we used it as reference for further experimentation. This concentration is 20-fold higher than the CS concentration present in human plasma and still did not exert a cytotoxic effect [26]. An increase in proliferation is observed in all conditions over time but no significant differences were found between the time points studied (data not shown). When comparing sources of origin, significant differences were found, after 24 h, for *S. canicula* and *R. clavata* CS, with improved proliferation regarding positive control (commercial bovine CS) and even though the significance is lost with incubation time, the trend is maintained. This was concordant for all concentrations. After 72 h, the two aforementioned sources, elicited a slight beneficial effect regarding both bovine CS and CS-free conditions, whilst metabolic activity in the presence of *P. glauca* CS decreased (significant vs. *S. canicula* at 10 and 50 µg/mL), albeit none was shown cytotoxic. Moreover, for the concentrations used, only *S. canicula* CS (200 µg/mL vs. 50 and 10 µg/mL) and bovine CS (200 and 100 µg/mL vs. 10 µg/mL), at 24 h, triggered a dose-dependent increase in cells’ metabolic activity, although this was not significantly sustained over time.

When comparing the metabolic tendency with MG-63 results, a similar response was obtained for 100 µg/mL concentration. Considering CS isomers, enrichment in 6S resulted in a decrease in proliferation amongst CS from fish sources. Moreover, when compared with 4S-enriched bovine CS, proliferation was improved in all fish sources, with significant differences found when comparing with *S. canicula* and *R. clavata* CS (24 h). So, in the case of T/C-28a2 cells, intermediate values of 6S led to improved proliferation. Even though the described immortalized cell lines were used for CS initial screening due to their availability regarding healthy human-derived primary cells, further validation in the latest would still be recommended.

### 2.3. Chondrogenesis of Bone Marrow-Derived Human Mesenchymal Stem Cells (MSCs)

**Figure 3:**
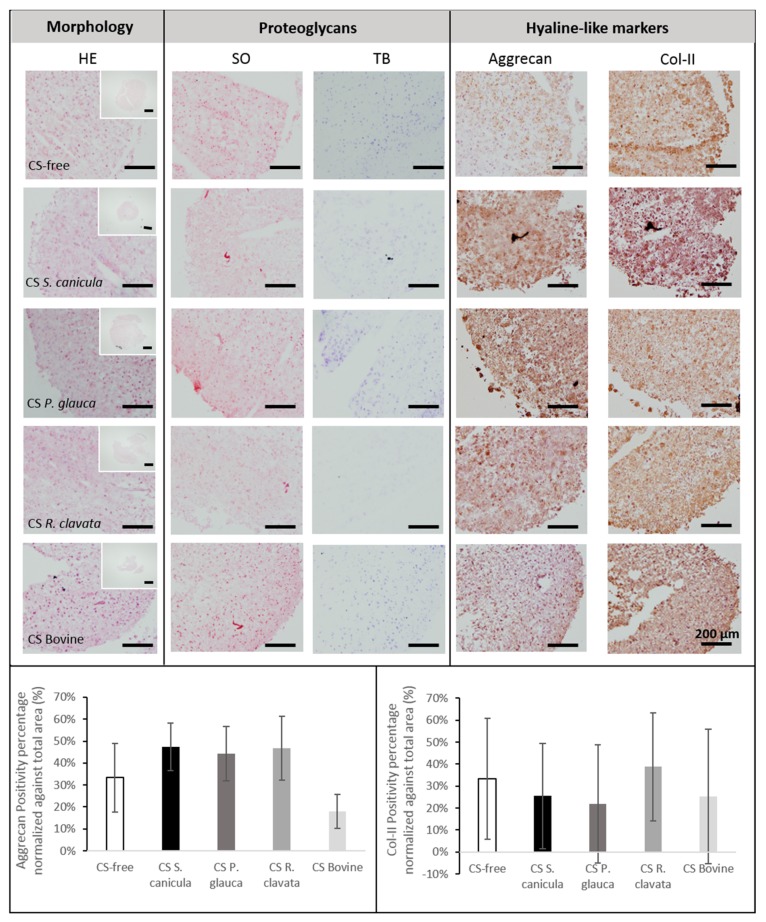
Histological analysis for morphology (haematoxylin and eosin, HE), proteoglycans (toluidine blue, TB) and sulfated glycosaminoglycans (GAGs) synthesis (safranin-O, SO), and immunolocalization of collagen-type II (Col-II) and aggrecan (scale bar = 200 µm), was performed in osteoarthritic bone marrow mesenchymal stromal cells (BM-MSCs) pellets, after 14 days, in chondrogenic medium supplemented with 100 µg/mL CS extracted from fish (*Prionace glauca*, *Raja clavata* and *Scyliorhinus canicula*) and bovine sources or CS-free. Immunopositive aggrecan (bottom, left) and collagen type-II (bottom, right) percentage area were normalized against cell-pellets total area (*n* = 3).

**Figure 4:**
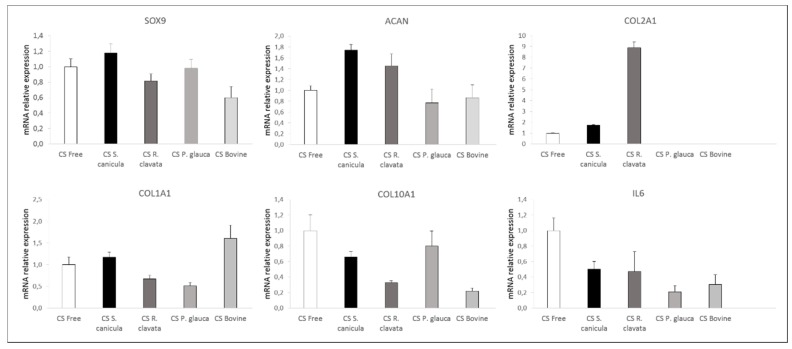
mRNA relative expression of *SOX9, ACAN, COL2A1, COL1A1, COL10A1* and *IL6* from osteoarthritic BM-MSCs pellets, after 14 days, in chondrogenic medium supplemented with 100 µg/mL CS extracted from fish (*Prionace glauca, Raja clavata* and *Scyliorhinus canicula*) and bovine sources. Data were normalized against chondrogenic medium in the absence of CS (CS-free) condition (positive control for chondrogenesis), represented as *n* = 1. Values are given as the mean of 4 donors with standard deviation. No significant differences were found.

We then investigated the effect of CS in chondrogenesis, by culturing bone marrow mesenchymal stromal cells (BM-MSCs), in a three-dimensional pellet conventional culture system, in chondrogenic medium stimulated with TGB-β_3_ and supplemented with 100 µg/mL CS from the different sources, during 14 days. When analyzing cell-pellets’ gross appearance, MSCs supplemented with *S. canicula* and bovine CS induced the formation of smaller sized pellets (Figure 3, small top HE). Chondrogenic differentiation was evaluated by analyzing the expression of genes (Figure 4) related to hyaline-like cartilage (*SOX9, ACAN* and *COL2A1*), fibrocartilage (*COL1A1*), hypertrophy (*COL10A1*) and inflammation (IL6) and the production of a sulfated-GAG- and proteoglycans-containing pellet matrix, by safranin-O and toluidine blue stainings (Figure 3). Conventional chondrogenic model condition (CS-free) led as expected to the expression of *SOX9, ACAN, COL2A1*. As reported in other models using bone marrow-derived MSCs [27], an inevitable transient tissue is formed, as observed by the expression of *COL10A1*.

When supplemented with CS, up-regulation of hyaline-like related markers was observed for *S. canicula* and *R. clavata*, which was also observed when compared to commercial bovine CS condition. *ACAN* expression was supported by correspondent protein synthesis (Figure 3). Even though the mRNA transcript level of the cartilage-related *COL2A1* gene was non-detectable, for *P. glauca* and bovine CS, col-II protein was found accumulated in the extracellular matrix (Figure 4). This might be explained by the fact that other mechanisms regulate the protein’s abundance. As mRNA is prone to degradation it is possible that mRNA has a rapid turnover while its protein has higher half-life and remains accumulated during the time studied [28]. Concomitantly, supplementation with these two CS sources led to a down-regulation in *COL1A1* (*R. clavata* vs. CS-free) and *COL10A1* (both vs. CS-free), indicating the formation of a more stable chondrogenic phenotype, whilst bovine CS presented the highest expression of *COL1A1* (vs. all other conditions), related to a fibrocartilage phenotype and *P. glauca* of *COL10A1* (vs. all conditions, except CS-free), indicative of a transient phenotype, resembling endochondral bone formation process, which can be detrimental for long-term articular cartilage restoration [13]. Moreover, the supplementation of CS from any source led to the down-regulation of inflammation marker IL6. Indeed, CS, had already shown in human chondrocytes stimulated with IL-1β to directly reduce inflammation by decreasing the presence of several protein complement components and also indirectly by increasing proteins such as TNFα-induced protein (TSG6) [29]. Nonetheless, none of the differences found were statistically significant. 

Overall, CS supplementation failed to exert a dramatic effect on both GAGs and proteoglycans (Figure 3, SO, TB), even though an increase in aggrecan synthesis was observed (Figure 3, top right and bottom left). In the absence of TGF-β and CS stimulation (negative control), no synthesis of aggrecan nor col-II was found (data not shown).

As many other authors, we support the use of MSCs isolated from OA patients in chondrogenic *in vitro* studies as opposed to those isolated from young healthy donors that may not reflect the expansion and differentiation characteristics of the disease condition [30,31]. However, even though MSCs derived from OA patients have shown their expansion and chondrogenic differentiation capacity, literature presents contradictory evidence in a variety of model systems and culture contexts for the chondrogenesis of human MSCs and this could be affecting our own results [32,33].

According to our results, the best chondrogenic potential was observed for *R. clavata* CS followed by *S. canicula* CS with intermediate 6S/4S, Ratio 1 = 0.46 ± 0.09; Ratio 2 = 0.67 ± 0.03 and Ratio 1 = 0.20 ± 0.07; Ratio 2 = 0.40 ± 0.03, respectively. Bovine CS exerted moderate chondrogenic promotor potential, although a fibrocartilage phenotype was observed due to an up-regulation of *COL1A1* expression. Although we could not find in the literature evidence on specific interactions between TGF-β_3_ and the isomers hereby reported, strong affinities have been reported between specific patterns of CS and members of the TGF-β family [18,34,35].

Moreover, other parameters such as the molecular weight (MW) of CS might also be playing a role in the proliferation and chondrogenic processes hereby described. Even though *P. glauca* CS presents higher 6S it also presents higher MW (60 kDa), compared to *R. clavata* (44 kDa) and *S. canicula* (43–45 kDa). Nonetheless, this would not apply when compared to bovine CS (close to 20 kDa) [19]. So, neither MW nor isomer prevalence seem to specifically correlate with an improved chondrogenesis. Even though the rationale behind this study was that a 6-S enrichment would improve both biological processes described, as supported by some authors [36], others have shown both an improvement in the proliferation and differentiation of human chondrocytes in CS-A 16 kDa vs. CS-C 34 kDa [37]. Another study with CS-A immobilized membranes promoted MSCs differentiation vs. no effect with CS-C [14]. Moreover, the current lack of understanding of CS mechanism of actions also supports further investigation [9]. In spite of the importance of CS sulfation, many sources used are not fully characterized and many studies do not specify the source used. Also, when performing analysis, authors use commercial CS-C derived from shark, which may proceed from endangered species. So, we hereby offer new insights for the use of other sources derived from fishery by-products, accessible under the European Landing Obligation, with potential applications in different OA treatment strategies.

## 3. Materials and Methods 

### 3.1. Chondroitin Sulfate

Isolated and purified samples (in powder) of chondroitin sulfate (CS) were obtained from piscine by-products sources, particularly *P. glauca* (head), *R. clavata* (skeleton) and *S. canicula* (fin). The procedure of CS extraction was previously described [38,39,40]. Commercially available CS from mammalian - bovine origin (Bioibérica S.A.) was used as positive reference. Molecular weight and correspondent ratios of CS-4 and CS-6 previously characterized are represented in Table 1 [19,22]. Prior to all cell culture experiments, CS from fish sources was washed three times with 70% ethanol. All dilutions, including those prepared for CS from bovine origin were filtered (0.22 µm).

### 3.2. Cellular Studies

#### 3.2.1. Viability in MG-63 Cell Line

MG-63 osteoblast-like cells (Sigma-Aldrich, St. Louis, MO, USA), were seeded at 3 × 10^3^ cells/mL, in minimum essential medium-Eagle with Earle’s balanced salt solution (EMEM, Lonza, Madrid, Spain) supplemented with 10% foetal bovine serum (FBS, Invitrogen, Madrid, Spain) and 1% antibiotic/ antifungal solution with penicillin, streptomycin, amphotericin B (Sigma), and incubated in 96-well plates (100 µL per well), at 37 °C, in a humidified atmosphere and 5% of CO_2_. After 24 h, growth media was replaced by fresh EMEM supplemented with the corresponding dilution of each CS, 50 µg/mL, 100 µg/mL, 0.5 mg/mL, 1 mg/mL and 10 mg/mL. MG-63 cells were maintained in culture for 72 h. Phenol solution at 6.4 mg/mL and tissue culture polystyrene (TCPS) were used as positive and negative controls for cytotoxicity, respectively. Three replicates per condition were evaluated and three independent experiments performed. After the incubation time, cytotoxicity was evaluated with the Cell Proliferation Kit I (MTT assay, Roche, Mannheim, Germany). This assay is based on the reduction of yellow tetrazolium salt MTT (3-(4,5-dimethyltyazolyl-2)-2,5-diphenyl tetrazolium bromide) to insoluble purple formazan crystals by the mitochondrial enzyme succinate dehydrogenase, which is only present in living cells. Briefly, supplemented growth medium (with diluted CS) was removed and fresh medium without FBS or antibiotic was added. Then, a volume of 10 μL of MTT labelling reagent in phosphate buffered saline (PBS) was also added to each well, for 4 h. After that, the formazan crystals formed were solubilized with 100 μL of 10% sodium dodecylsulfate (SDS) in 0.01 M HCl. The plate was incubated overnight and quantified at 595 nm and 655 nm. The optical density data were normalized against the values obtained for the CS-free condition, which was assigned the value of 1.

#### 3.2.2. Viability and Proliferation in T/C-28a2 Cell Line

Human chondrocytes cell line (T/C-28a2) was expanded until confluence, under basal medium (Dulbecco’s modified Eagle’s medium (DMEM), Lonza, Madrid, Spain) supplemented with 10% foetal bovine serum (FBS, Invitrogen, Madrid, Spain) and penicillin/streptomycin (10,000 IU/mL) (Sigma-Aldrich, Madrid, Spain). Cells were then seeded in a 96-well tissue culture microplate, under the same medium with a density of 3000 cells per well (*n* = 5), repeated 3 times. 24 h later, culture medium was replaced by basal medium supplemented with different concentrations, 10, 50, 100 and 200 µg/mL of fish- or bovine-origin CS as well as CS-free conditions (or TCPS). Cell proliferation was quantitatively measured by the reduction percentage of alamar blue (ThermoFisher, Eugene, OR, USA) after 24, 48 and 72 h, according to the manufacturer’s instructions. The assay reveals the metabolic activity of cells by detecting mitochondrial activity. However, rather than obtaining endpoint readings such as when using other conventional methods based on the cleavage of tetrazolium salts, like MTT, it allows the follow-up of the same set of cells in a kinetic fashion. For each time period, cells were incubated for 3 hours with 10% alamar blue prior to absorbance reading, at 570 nm and 600 nm. The intensity of the blue color obtained is directly proportional to the metabolic activity of cell populations. Blank values were subtracted to exclude background activity.

#### 3.2.3. Chondrogenesis of Bone Marrow-Derived Human MSCs 

Human mesenchymal stromal stem cells (MSCs) were isolated from the bone marrow (BM) of femur heads obtained from 4 osteoarthritic (OA) donors (2 females and 2 males, with ages comprised between 65 and 89). Human tissue samples used belonged to the Sample Collection for Research on Rheumatic Diseases authorized by the Galician Research Ethics Committee (CAEIG) with registry code 2013/107 and inscribed in the National Registry of Biobanks, Collections Section code C.0000424. The patients signed an informed consent agreement form prior to collection. The study was conducted in accordance with the Declaration of Helsinki and the protocol was approved by CAEIG (Project Identification Code: 2018/267). BM-MSCs were extracted by washing the bone marrow with 50 mL basal medium composed of DMEM supplemented with 20% FBS and penicillin/streptomycin (10,000 IU/mL) (Sigma-Aldrich, Madrid, Spain). The resulting cell suspension was filtered through a sterile 40 µm filter and centrifuged at 300 g for 8 minutes. Then, cells were cultured in basal medium at a density of 1 × 10^5^ cells/cm^2^, in a 5% humidified CO_2_ atmosphere, at 37 ºC, until 90% confluent. Pre-plating technique was performed to avoid any remaining fibroblasts [41]. Cells used in all experiments were mycoplasma-free. BM-MSCs (Passage 3) were seeded under pellet three-dimensional culture system. Briefly, 2.5 × 10^5^ cells, were suspended in 500 μL of chondrogenic differentiation medium (CM), composed of DMEM supplemented with knockout serum (15%), ascorbic acid (10 µL/mL), transferrin (6 µL/mL), dexamethasone (10 µM), retinoic acid (10–7 M), and transforming growth factor beta 3 (10 ng/mL), centrifuged at 600 g, for 10 min, in 15 mL polypropylene conical tubes and incubated under 37 °C and 5% CO_2_. After 48 h, medium was replaced by fresh CM supplemented with 100 µg/mL CS from both fish and bovine origin sources. DMEM 20% was used as a negative control of chondrogenesis. Cells were kept until day 14, with medium replaced every 2–3 days. Three replicates were included for each condition and four independent experiments performed.

#### 3.2.4. Molecular Expression

Total RNA was isolated from cell pellets. Briefly, pellets frozen under liquid nitrogen were pulverized in a micro-dismembrator (Retsch MM200, Hann, Germany) and then transferred to RNAse-free tubes containing TRIzol (Invitrogen, Carlsbad, CA, USA). After digestion, the aqueous phase containing RNA was separated by the addition of chloroform. RNA was precipitated with isopropanol and then washed in 75% ethanol. Finally, pellets were air-dried and re-dissolved in 10 µL RNase-free water. RNA concentrations were determined by measuring the absorbance at 260 nm with a nanodrop spectrophotometer (Fisher Scientific, Wilmington, DE, USA,). RNA samples were converted into cDNA using the SuperScript VILO synthesis kit (Invitrogen). Gene expression was determined by real-time reverse transcription polymerase chain reaction (qRT-PCR), conducted in a LightCycler 480 Instrument (Roche) using the LightCycler 480 Probes Master protocol. Amplification of mRNA was performed using custom primers for aggrecan (*ACAN*), sex-determining region Y-box 9 (*SOX9*), interleukin 6 (*IL6*), collagens type-II (*COL2A1*), -I (*COL1A1*), and -X (*COL10A1*), as shown in Table 2. Ribosomal protein L13a (*RPL13a*) was used as housekeeping gene (HKG). Quantitative RT-PCR data analysis was performed using qbase+ software, version 3.0 (Biogazelle, Zwijnaarde, Belgium). Relative levels of expression were calculated and normalized against the values obtained for the CS-free condition for each gene, which was assigned the value of 1.

#### 3.2.5. Histology and Immunohistochemistry

Following chondrogenic differentiation, pellets were frozen in OCT embedding matrix (Sakura). Histological staining was performed with haematoxylin and eosin (HE), safranin-O (SO) and toluidine blue (TB). Immunohistochemical labeling was performed for monoclonal antibodies for collagen type-II (col-II) (1:50, Abcam) and aggrecan (1:100, Abcam) in cryosections of 4 µm of each pellet. Secondary antibodies were detected using a polymer-labelled HRP complex (EnVision Detection System Peroxidase/DAB Kit, DAKO). The samples were analyzed using a Leica DM 200 Led light microscope (Leica Microsystems, Madrid, Spain) equipped with a Leica digital camera (Model ICC50W), at magnifications of 4× and 20×. The percentage of immunopositive area was calculated with ImageJ software (version 1.51K, U. S. National Institutes of Health, Bethesda, MD, USA) and normalized against pellet area.

#### 3.2.6. Statistical Analysis

All data presented were expressed as mean values ± standard deviation (SD). All experiments were performed in triplicate, unless stated. Data obtained from the MG-63 cell line incubated with different concentrations of CS from fish and bovine sources were normalized against CS-free. Each element was analyzed using the non-parametric Mann–Whitney statistical test. For cell viability, molecular biology and immunopositive areas percentage, one-way analysis of variance (ANOVA) and Bonferroni’s multiple comparison were performed. Statistical differences were evaluated using the R Open 3.5.3. statistical software with a significance level of *p* < 0.05.

## 4. Conclusions

In summary, intermediate values of both isomeric CS-4 and CS-6, from *S. canicula* and *R. clavata* species, contributed to significantly improve osteoblastic and chondrocytic cell lines’ proliferation whilst prevalence of CS-6, present in *P. glauca*, failed to exert major effects for the time points studied. Moreover, the sulfation patterns of exogenous addition of CS discretely affected MSCs chondrogenic differentiation and, even though both *S. canicula* and *R. clavata* CS showed improved chondrogenic-related markers expression, these were not statistically significant.

## Figures and Tables

**Table 1:** CS-4 and CS-6 ratios and corresponding molecular weight (MW) determined for CS from bovine and piscine sources, as previously determined.

	Bovine	*P. glauca*	*R. clavata*	*S. canicula*
Ratio 1* (CS-4)	0.8 ± 0.2	0.27 ± 0.07	0.46 ± 0.09	0.20 ± 0.07
Ratio 2* (CS-6)	0.22 ± 0.01	0.96 ± 0.09	0.67 ± 0.03	0.40 ± 0.03
MW**	≈ 20 kDa	60 kDa	44 kDa	43–45 kDa

Index for CS-4 prevalence: Ratio 1= 0.8–1.0 and Ratio 2= 0.12–0.26; Index for CS-6 prevalence: Ratio 1 = 0.2–0.4 and Ratio 2= 0.8–1.0. References: * [22] ** [19].

**Table 2:** List of primers used for real-time reverse transcription polymerase chain reaction (qRT-PCR).

Gene	Forward	Reverse	Probes	Gene Bank A. Number
*RPL13a*	CAAGCGGATGAACACCAAC	TGTGGGGCAGCATACCTC	28	NM_012423.2
*SOX9*	GTACCCGCACTTGCACAAC	TCGCTCTCGTTCAGAAGTCTC	61	NM_000346
*ACAN*	CGGTCTACCTCTACCCTAACCA	GAGAAGGAACCGCTGAAATG	38	NM_013227.3
*COL1A1*	CTGGCCCCATTGGTAATGT	ACCAGGGAAACCAGTAGCAC	1	NM_000088.3
*COL2A1*	TGGTGCTAATGGCGAGAAG	CCCAGTCTCTCCACGTTCAC	4	NM_001844.4
*COL10A1*	CACCTTCTGCACTGCTCATC	GGCAGCATATTCTCAGATGGA	6	NM_000493.3
*IL6*	GATGAGTACAAAAGTCCTGATCCA	CTGCAGCCACTGGTTCTGT	40	NM_000600.4
